# Imaging reconstruction comparison of different ghost imaging algorithms

**DOI:** 10.1038/s41598-020-71642-2

**Published:** 2020-09-03

**Authors:** Hong-Chao Liu

**Affiliations:** 1 Joint Key Laboratory of the Ministry of Education, Institute of Applied Physics and Materials Engineering, University of Macau, Avenida da Universidade, Taipa, Macao SAR China; 2 Faculty of Science and Technology, University of Macau, Avenida da Universidade, Taipa, Macao SAR China

**Keywords:** Optics and photonics, Physics

## Abstract

As an indirect and computational imaging approach, imaging reconstruction efficiency is critical for ghost imaging (GI). Here, we compare different GI algorithms, including logarithmic GI and exponential GI we proposed, by numerically analysing their imaging reconstruction efficiency and error tolerance. Simulation results show that compressive GI algorithm has the highest reconstruction efficiency due to its global optimization property. Error tolerance studies further manifest that compressive GI and exponential GI are sensitive to the error ratio. By replacing the bucket input of compressive GI with different bucket object signal functions, we integrate compressive GI with other GI algorithms and discuss their imaging efficiency. With the combination between the differential GI (or normalized GI) and compressive GI, both reconstruction efficiency and error tolerance will present the best performance. Moreover, an optical encryption is proposed by combining logarithmic GI, exponential GI and compressive GI, which can enhance the encryption security based on GI principle.

## Introduction

As an indirect imaging technique, ghost imaging (GI) obtains the object image from the correlation of light intensity fluctuation correlation. Usually, two beams are required in the GI process: One, called object beam, interacts with object and is bucket detected by a single-pixel camera; the other, called reference beam, carries no object information and is detected by a spatially resolved multi-pixel detector. Since the first GI experiment was reported in 1995^1^, GI attracted considerable research interests^2–21^ both in fundamental physics, such as the EPR paradox study^7^, and practical applications, such as turbulence-free detection^8,10^ and medical imaging^16–18^. Nevertheless, the correlation measurement of GI always requires lots of integration time, which is the bottleneck blocking its wide applications. In order to enhance the imaging efficiency and quality, different GI reconstruction algorithms were proposed, including high-order ghost imaging (HGI)^22–26^, differential ghost imaging (DGI)^27^, normalized ghost imaging (NGI)^28^, compressive ghost imaging (CGI)^29^, etc. As these GI algorithms have completely different definitions, a comparison is necessary to demonstrate their performance in imaging reconstruction. Moreover, a combination between different GI algorithms is lack of investigation so far, least of all for relevant potential applications.

In this work, we compare imaging reconstruction efficiency and error tolerance of six representative GI algorithms, together with logarithmic GI and exponential GI we proposed^30^. Numerical simulations show that compressive GI algorithm takes a great advantage in the imaging reconstruction process due to its global optimization property. Moreover, error tolerance studies manifest that compressive GI and exponential GI are sensitive to the error ratio. By further replacing the bucket input of compressive GI with different bucket object signal functions, we combine compressive GI with other seven GI algorithms and discuss their imaging efficiency. With a combination of differential GI (or normalized GI) and compressive GI, a high reconstruction efficiency and error tolerance algorithm can be achieved. In addition, by combining logarithmic GI, exponential GI and compressive GI, we propose an optical encryption which can enhance the security in the encryption process.

## Results

### Comparison of imaging efficiency of different ghost imaging algorithms

**Figure 1:**
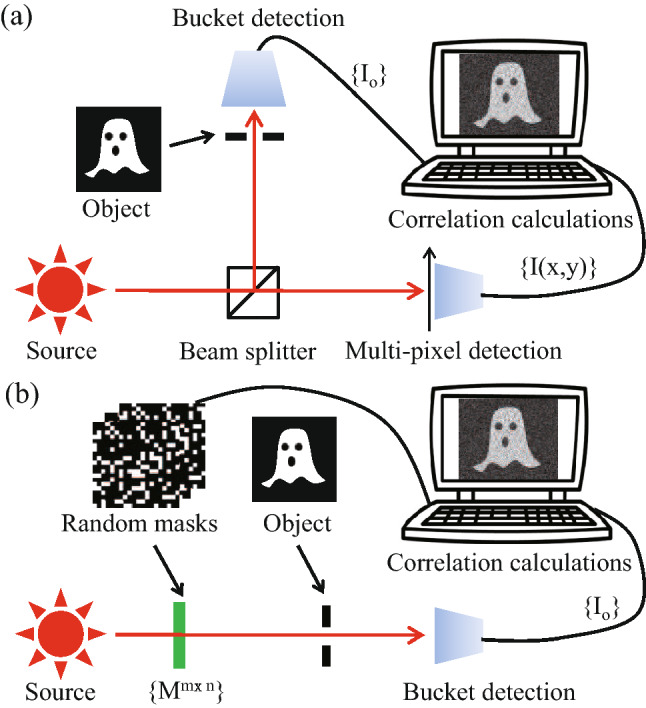
Schematic setup of (**a**) traditional two-detector ghost imaging and (**b**) computational ghost imaging.

Figure 1(a,b) show the schematic setup of traditional two-detector GI and computational GI, respectively. In traditional two-detector GI, the illuminating patterns from the source are passive ones which are always random and non-deterministic. In computational GI, the illuminating patterns are computer-generated where deterministic patterns become possible^9,14,31,32^. By calculating the correlation between the bucket object signals and illuminating patterns, the ghost image can be reconstructed. Reconstruction algorithms and illuminating patterns are two main factors give significant impacts on GI efficiency. Compared with the random patterns, different deterministic patterns (e.g., Hadamard^33–35^ and Fourier^36^ patterns) have been proposed and demonstrated their advantages in computational GI configuration with a low sampling ratio due to their characteristic (e.g., orthogonality). Nevertheless, the advantages of deterministic patterns are always accompanied by some limitations (e.g., the inapplicability in passive illumination cases, the size limitation of Hadamard matrix and the order effect of Hadamard patterns^34,35^), which complicates the pattern effect on GI efficiency. For simplicity and without loss of generality, we here choose random patterns to focus on the study of GI algorithm effect. Eight different GI algorithms are compared below, and their definitions can be found in the Method Section.

**Figure 2:**
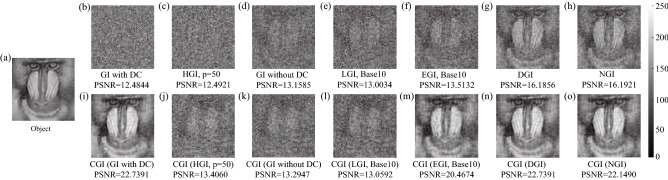
Simulated ghost images with different algorithms: (**a**) Object, (**b**) ghost imaging with DC background, (**c**) high-order ghost imaging, (**d**) ghost imaging without DC background, (**e**) logarithmic ghost imaging, (**f**) exponential ghost imaging, (**g**) differential ghost imaging, (**h**) normalized ghost imaging, (**i**)–(**o**) compressive ghost imaging with different bucket object signal function $F(Io_i)$ in (**b**)–(**h**), respectively. The measurement number $N=10000$ for all algorithms.

Figure 2 shows the reconstructed ghost images with different GI algorithms in simulations. A grayscaled Baboon picture ($101\times 101$ pixels) acts as the imaging object, as shown in Fig. 2(a). In the simulation, we set $p=50$ and $q=1$ in HGI algorithm, because a large *p* and a small *q* will largely increase the image visibility and suppress the noise level^25,26^. For both LGI and EGI algorithms, we set base $A,B=10$ and constant $C=1$ in the simulation. The measurement number *N* is 10,000 for all algorithms in the simulations. Peak signal-to-noise ratio (PSNR) is applied here to evaluate the image quality below, which is defined as

1 $$\begin{aligned} \text{ PSNR }=10\text{ log}_{10}\left( \dfrac{\text{ MAX}^2}{\text{ MSE }}\right) , \end{aligned}$$

where MAX=255 is the maximum possible pixel value of the image. MSE is the mean square error, read by $\dfrac{1}{m\times n}\sum _{i,j}\left[ T_{re}(x_i,y_j)-T(x_i,y_j)\right] ^2$, where $T_{re}(x_i,y_j)$ and $T(x_i,y_j)$ are the pixel values of the recovered image and the object, respectively.

**Figure 3:**
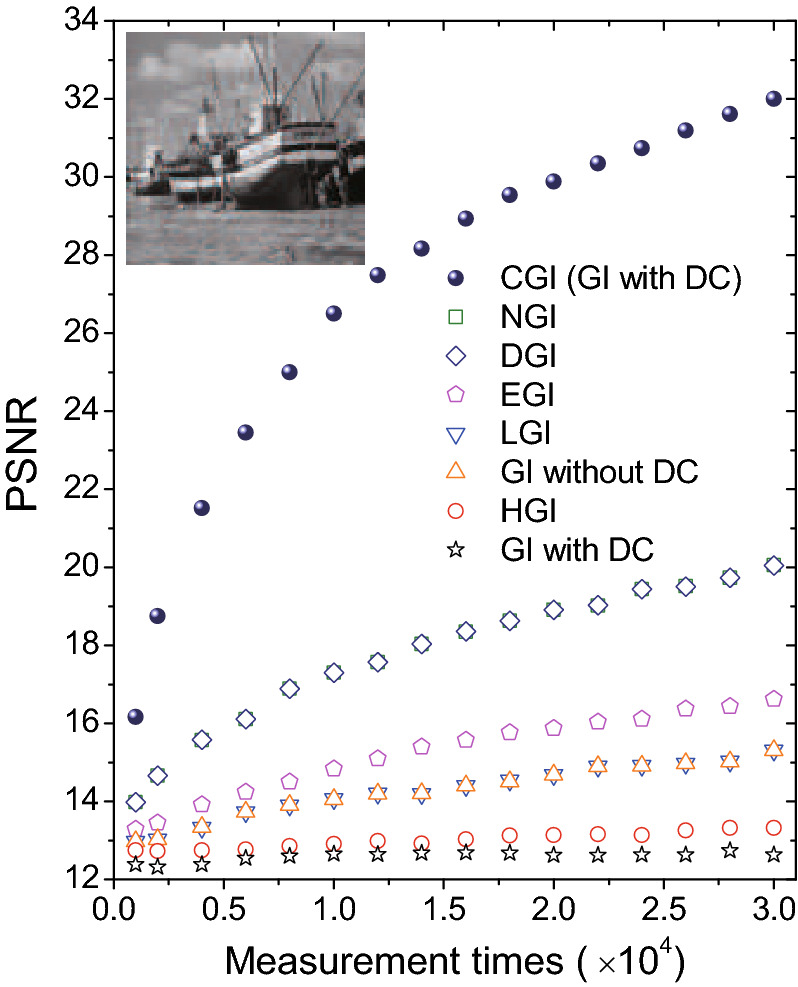
Comparison of different ghost imaging algorithms in simulations. The inset grayscaled boat picture ($101\times 101$ pixels) acts as the object.

Figure 2(b–i) show a comparison of eight different GI algorithms introduced above. Simulation results manifest that GI with DC component and HGI algorithms provide no object information, indicating that neither of them can overcome the Nyquist limit ($N=101\times 101$). A little improvement is achieved when one chooses the GI algorithm without DC component or LGI algorithm as shown in Fig. 2(d,e). Although EGI offers a better performance than former four algorithms, it fails to present a clear image structure as DGI and NGI do in Fig. 2(g,h). With a great advantage of the global optimization, CGI in Fig. 2(i) recovers nearly all details of the object image within the Nyquist limit. To clearly demonstrate the recovery efficiency of different GI algorithms, Fig. 3 is plotted with a grayscaled boat picture ($101\times 101$ pixels) acting as the object. PSNR values show that CGI can recover an image with the quality comparable to the ones of DGI and NGI by performing one order less measurements. Meanwhile, DGI and NGI take an advantage over the EGI, LGI and GI without DC component algorithms. GI with DC component and HGI algorithms conduct the simplest calculations in the imaging process, nevertheless, at the expense of the lowest imaging efficiency.

**Figure 4:**
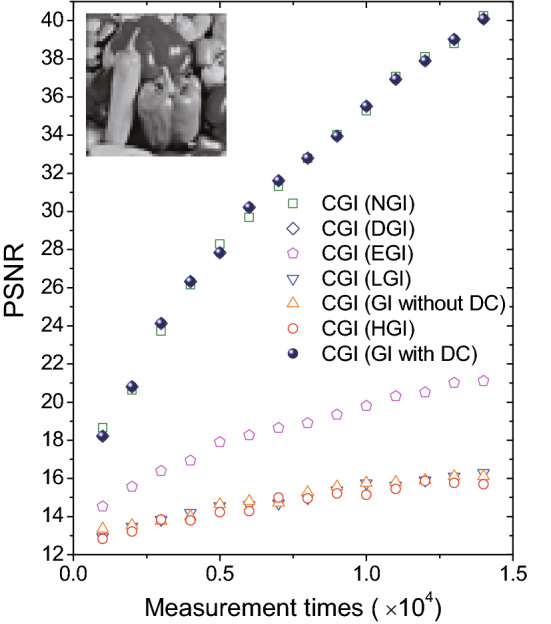
Comparison of compressive ghost imaging with different bucket object signal function $F(Io_i)$ in simulations. The inset grayscaled peppers picture ($51\times 51$ pixels) acts as the object.

Because CGI has its unique merit in the imaging reconstruction calculation as demonstrated above, we further apply different $F(Io_i)$ in other algorithms to replace the bucket object signal $Io_i$ of the CGI as the input, and discuss their imaging efficiency. Figure 2(i–o) show a comparison of CGI with seven different bucket object signal functions $F(Io_i)$. One can see that CGI with $F(Io_i)=Io_i$ and $F(Io_i)=Io_i-X_i\left\langle {Io} \right\rangle /\left\langle X\right\rangle$ take exactly the same PSNR values as shown in Fig. 2(i) and Fig. 2(n). More generally, one can prove that when $F(Io_i)=c_1 Io_i+c_2 X_i$ ($c_1$ and $c_2$ are constant, $c_1\ne 0$), CGI calculation will provide the same PSNR value as the one with $F(Io_i)=Io_i$, indicating that the bucket reference signal $X_i$ brings no effect on the orthogonal matching pursuit method applied in the CGI simulations. Meanwhile, as shown in Fig. 2(o), CGI with $F(Io_i)=Io_i/X_i-\left\langle {Io} \right\rangle /\left\langle X\right\rangle$ can also achieve a high imaging efficiency comparable to the DGI and GI with DC cases in Fig. 2(i) and Fig. 2(n). In addition, CGI with bucket object signal of HGI, GI without DC component and LGI, show low recovery efficiencies, but EGI offers a medium imaging quality, as shown in Fig. 2(j–m). In order to quantitatively estimate the efficiency of CGI with different bucket object signal functions, Fig. 4 is plotted. A grayscaled peppers picture ($51\times 51$ pixels) plays the role of object. As can be seen, CGI with bucket object signal functions of DGI, NGI and GI with DC component always achieve a high imaging efficiency, in comparison to the medium efficiency of CGI (EGI) case and other three low efficiency cases, which is consistent with the results in Fig. 2.

In addition, it should be mentioned that, in the definition of Eq. (3), the DC component of the reference beam is sometimes removed together with the DC component of the object beam^27,28^, leading Eq. (3) into the expression as $G^{(2)}=(1/N) \sum _{i=1}^{N}(Io_{i}-\left\langle Io \right\rangle ) (I_{i}(x,y)-\left\langle X \right\rangle )$. With this definition, the imaging efficiency of GI without DC component algorithm will keep unchanged. However, the efficiency of CGI (GI without DC) case will be improved as high as the one of CGI (GI with DC) case.

### Comparison of error tolerance of different ghost imaging algorithms

**Figure 5:**
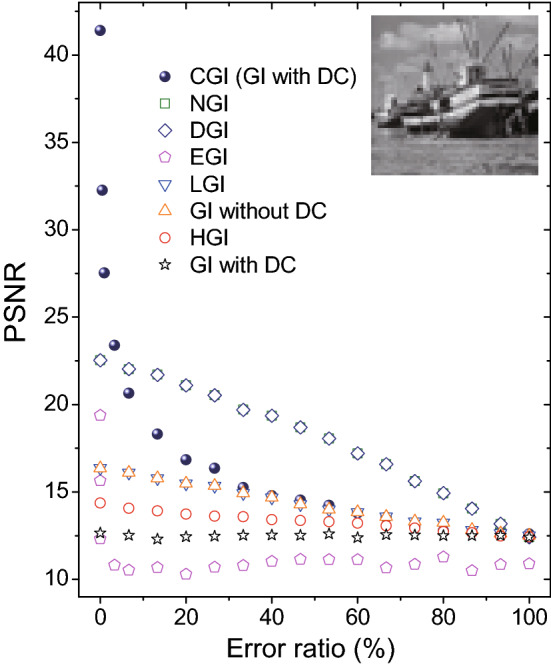
Comparison of error tolerance of different ghost imaging algorithms in simulations. The inset grayscaled boat picture ($51\times 51$ pixels) acts as the object. The measurement number *N* is 15,000.

To further compare the reconstruction efficiency, the error tolerance of different GI algorithms are discussed below. We here introduce the error by messing up the order *i* of reference signals (or random matrix $M_i^{m\times n}$). In Fig. 5, one can see that CGI (GI with DC) algorithm shows a dramatic decrease as the error ratio increases although it has the highest recovery efficiency with no error. When the error ratio is greater than 10$\%$, the imaging quality of CGI (GI with DC) is lower than DGI and NGI algorithms. As the error ratio increases more than 30$\%$, the imaging quality of CGI (GI with DC) becomes comparable to the ones of GI without DC component and LGI algorithms. It implies that a global optimization algorithm is sensitive to the error. More sensitive than the CGI (GI with DC) algorithm, EGI fails to recover the image information even with 5$\%$ error ratio, as shown in Fig. 5. This might be caused by its divergence reconstruction calculations, that is, the exponential function will largely amplify the input errors. By contrast, all other algorithms exhibit nearly linear decrease as the error ratio increases, as shown in Fig. 5.

**Figure 6:**
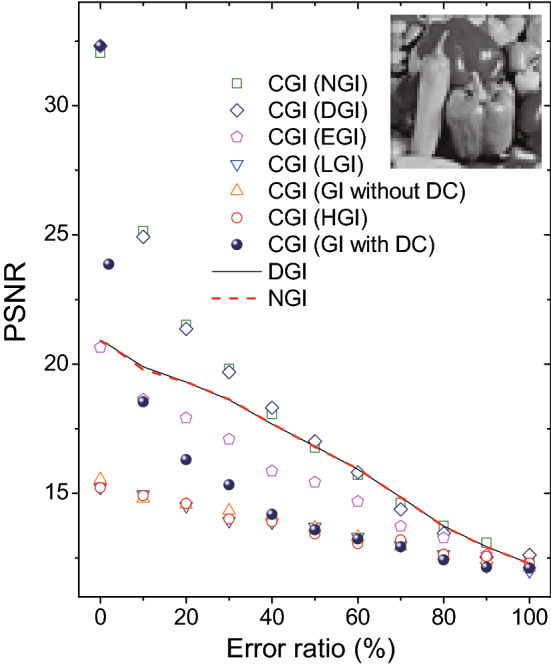
Comparison of error tolerance of compressive ghost imaging with different bucket object signal function $F(Io_i)$ in simulations. The inset grayscaled peppers picture ($51\times 51$ pixels) acts as the object. The measurement number *N* is 10,000.

Moreover, the error tolerance of CGI algorithm with different bucket object signal function $F(Io_i)$ are studied in simulations. In Fig. 6, three cases, i.e., CGI (GI with DC), CGI (DGI) and CGI (NGI), have similar PSNR values when error ratio is 0$\%$. Nevertheless, the recovery efficiency of CGI (GI with DC) decreases much faster than the other two cases. Interestingly, although both CGI (GI with DC) and EGI are extremely sensitive to the error as discussed in Fig. 5, the combination of them, i.e., CGI (EGI) are more robust than the CGI (GI with DC) case. When the error ratio is greater than 10$\%$, the imaging quality of CGI (EGI) becomes higher than the CGI (GI with DC) case. The solid and dash lines in Fig. 6 show the DGI and EGI algorithms, respectively. Comparison manifests that both DGI and NGI have better performance than other algorithms except CGI (DGI) and CGI (NGI), when taking the error into consideration. Therefore, CGI (DGI) and CGI (NGI) are two best choices for GI reconstruction whatever error level it is.

### Optical encryption scheme based on the combination of different ghost imaging algorithms

The imaging principle of GI offered an optical encryption scheme^9^, where the bucket object signals of target information were viewed as the ciphertext and random matrices played the role of keys. Based on this scheme, different optical encryption methods were developed, such as gray-scale and color image encryption^37^, multiple-image encryption^38^, metasurface-based encryption^39^, specific phase masks encryption^40^, symmetric-asymmetric cryptography^41^, etc. Different from above methods, we here propose an optical encryption scheme based on the combination of different GI algorithms, where the bucket object signals of GI are re-encoded into different bucket object signal functions as the ciphertext. The combinations of different bucket object signal functions and their relevant parameters (e.g., base value of LGI and EGI) will protect the GI information against the eavesdropper.

**Figure 7:**
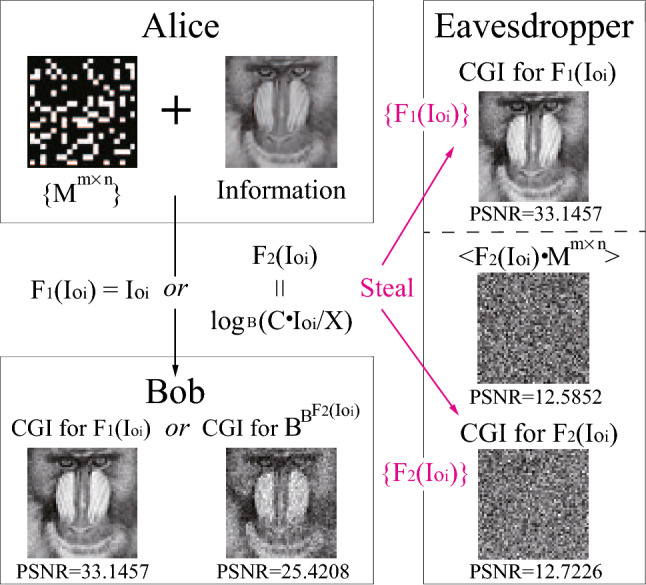
Scheme of the encryption method based on logarithmic ghost imaging and exponential ghost imaging. The object is a $51\times 51$ pixels grayscaled baboon picture. The ghost images are reconstructed with 15,000 measuring times, and $B=10$, $C=1$ in the simulations.

The encryption scheme is shown in Fig. 7. Suppose Alice plans to send a baboon picture ($51\times 51$ pixels) to Bob. By employing the computational GI experimental setup, Alice encodes the picture into a series of numbers $F_1(Io_i)=Io_i$. Using the shared dictionary, i.e., the random matrices $\{ M^{m\times n}\}$, Bob therefore can recover the information by using any GI algorithms, as shown in Fig. 7. Assuming there is an eavesdropper who has stolen the shared dictionary $\{ M^{m\times n}\}$ together with the number series $F_1(Io_i)=Io_i$. Obviously, the eavesdropper can easily decode the information by using the most-efficient algorithm CGI. In order to ensure Bob be able to obtain the picture and simultaneously keep the information safe, a combination of different GI algorithms is a good option. Here, Alice can choose LGI and EGI to improve the security. As shown in Fig. 7, Alice encodes the number series into the form of $F_2(Io_i)=\mathrm{{log}}_B (C \cdot Io_i/X)$. After receiving the message from Alice, Bob can apply $B^{B^{F_2(Io_i)}}$ to the CGI algorithm to decode the picture. Without knowing the combination form of $F_2(Io_i)$, the eavesdropper is unable to decrypt the information even he (or she) steals all shared dictionary and number series by using the CGI or other reconstruction algorithm, as shown in Fig. 7. Therefore, a combination of different GI algorithms can provide additional security lock to the optical encryption process.

## Conclusion

In summary, we compared the imaging reconstruction efficiency and error tolerance of different GI algorithms. Simulations based on the computational GI scheme have manifested that CGI algorithm has the highest reconstruction efficiency due to its global optimization property. The imaging efficiency of compressive GI with different bucket object signal functions has also been discussed. Error tolerance studies have further demonstrated that CGI and EGI are sensitive to the error ratio. With DGI or NGI bucket object signal function as the input in the CGI algorithm, the imaging reconstruction efficiency would be the highest one whatever error level it is. In addition, an optical encryption was proposed by combining different GI algorithms. The combination of LGI, EGI and CGI can enhance the security of the GI encryption process.

## Methods

### Definitions of different ghost imaging algorithms

The traditional second-order GI reconstruction algorithm is expressed as

2 $$\begin{aligned} G^{(2)}=\dfrac{1}{N} \sum _{i=1}^{N}Io_{i} I_{i}(x,y), \end{aligned}$$

where $Io_{i}$ and $I_{i}(x,y)$ represent the bucket intensity signal of object beam and the spatial intensity distribution of reference beam in the *i*th measurement, respectively. To remove the background effect and achieve a better GI quality, a DC component is usually subtracted from the bucket object signal, leading the GI algorithm into the form as

3 $$\begin{aligned} G^{(2)}=\dfrac{1}{N} \sum _{i=1}^{N}(Io_{i}-\left\langle Io \right\rangle ) I_{i}(x,y), \end{aligned}$$

where $\left\langle \cdots \right\rangle$ denotes an ensemble average for *N* measurements. Based on the two-detector experimental setup, the most-used form of HGI algorithm is given as^23,25,26^

4 $$\begin{aligned} G^{(p,q)}=\dfrac{1}{N} \sum _{i=1}^{N}(Io_{i})^p (I_{i}(x,y))^q, \end{aligned}$$

where *p* and *q* are the power indices of the bucket object signal and reference signal, respectively.

Developing from the basic GI definition in Eq. (2), in DGI and NGI algorithms, the bucket object signal $Io_{i}$ is replaced by different bucket object signal functions $F(Io_{i})$. In DGI algorithm, the bucket object signal function is defined as^27^

5 $$\begin{aligned} F(Io_{i})_{DGI}=Io_i-\dfrac{\left\langle {Io} \right\rangle }{\left\langle X\right\rangle }X_i, \end{aligned}$$

where $X_i=\int I_{i}(x,y)dxdy$ is the total intensity of the reference beam in the *i*th measurement. In NGI algorithm, the bucket object signal function is defined as^28^

6 $$\begin{aligned} F(Io_{i})_{NGI}=\dfrac{Io_i}{X_i}-\dfrac{\left\langle {Io} \right\rangle }{\left\langle X\right\rangle }. \end{aligned}$$

In analogy to the definitions of DGI and NGI, we recently proposed the LGI and EGI algorithms by defining the bucket object signal as logarithmic and exponential functions of $Io_{i}$, respectively^30^. Thus, the reconstruction algorithm of LGI is

7 $$\begin{aligned} G^{log}=\dfrac{1}{N} \sum _{i=1}^{N}\left( \mathrm{{log}}_A\dfrac{Io_{i}}{\left\langle {Io} \right\rangle }\right) I_{i}(x,y), \end{aligned}$$

where *A* is the base of the logarithmic function. The reconstruction algorithm of EGI is expressed as

8 $$\begin{aligned} G^{exp}=\dfrac{1}{N} \sum _{i=1}^{N}{B}^{C\cdot (Io_{i}/X_i)} I_{i}(x,y), \end{aligned}$$

where *B* is the base of the exponential function, *C* is a constant depending on the value of base *B*.

Different from all algorithms above, compressive sensing is an iterative algorithm with global optimization based on the sparsity of the imaging object^29,42^. By applying compressive sensing to the GI reconstruction process, the two-dimensional reference signal $I_{i}(x,y)$ are resized into a row vector ($1\times K$, $K=m\times n$), where *m* and *n* are the pixel numbers of the *x* and *y* directions, respectively. And the set $\{I_{i}(x,y)\}$ of *N* measurements is rewritten into a two-dimensional matrix *D* ($N\times K$). Meanwhile, the set of bucket object signal $\{Io_{i}\}$ is expressed as a column vector $I^{CGI}$ ($N\times 1$). If the object is sparse in matrix *D*, its image can be reconstructed by solving the convex optimization program as^11,29,42^

9 $$\begin{aligned} T^{CGI}=\mid T\mid ,\ \text{ min }\parallel T\parallel _{1} \text{ subject } \text{ to } I^{CGI}=DT, \end{aligned}$$

where $T^{CGI}$ is the recovered image information, *T* is the imaging object information, and $\parallel T\parallel _{1}$ is the $L_1$-norm of *T*. All GI algorithms above can also work well within both the traditional two-detecor GI and computational GI scheme shown in Fig. 1.

### Information for target images and random matrices

All image targets used in this work were grayscaled with 8-bit. In the simulations, the pixel values of all random matrices $\{M^{m\times n}\}$ ranged from 0 to 1 with average value as 0.5. The widely-used orthogonal matching pursuit method were employed in the CGI simulation program. In the error tolerance simulations, we introduced the error by messing up the order *i* of random matrix $M_i^{m\times n}$. 5$\%$ error ratio means 5$\%$ bucket signals had a random combination with random matrices $\{M^{m\times n}\}$, whereas the rest 95$\%$ bucket signals were well matched with their corresponding random matrices $\{M^{m\times n}\}$.
